# Development and validation of platelet-to-albumin ratio as a clinical predictor for diffuse large B-cell lymphoma

**DOI:** 10.3389/fonc.2023.1138284

**Published:** 2023-06-08

**Authors:** Jinghan Wang, Linjie Li, Fang Yu, Junyu Zhang, Liping Mao, Bocheng Chen, Xuelian Hu, Hongmei Zhou, Wanzhuo Xie, Hongyan Tong, Jie Jin

**Affiliations:** ^1^ Department of Hematology, The First Affiliated Hospital, Zhejiang University School of Medicine, Hangzhou, China; ^2^ Department of Hematology, Lishui Municipal Central Hospital, Lishui, China; ^3^ Department of Pathology, The First Affiliated Hospital of Zhejiang University School of Medicine, Hangzhou, China; ^4^ Department of Hematology, Yongkang First People’s Hospital Affiliated to Hangzhou Medical College, Yongkang, China

**Keywords:** diffuse large B-cell lymphoma, U-shaped relationship, survival, clinical parameters, platelet counts

## Abstract

**Introduction:**

Diffuse large B-cell lymphoma (DLBCL) is the most common subtypes of lymphoma. Clinical biomarkers are still required for DLBCL patients to identify high-risk patients. Therefore, we developed and validated the platelet-to-albumin (PTA) ratio as a predictor for DLBCL patients.

**Methods:**

A group of 749 patients was randomly divided into a training set (600 patients) and an internal validation set (149 cases). The independent cohort of 110 patients was enrolled from the other hospital as an external validation set. Penalized smoothing spline (PS) Cox regression models were used to explore the non-linear relationship between the PTA ratio and overall survival (OS) as well as progression-free survival (PFS), respectively.

**Results:**

A U-shaped relation between the PTA ratio and PFS was identified in the training set. The PTA ratio less than 2.7 or greater than 8.6 was associated with the shorter PFS. Additionally, the PTA ratio had an additional prognostic value to the well-established predictors. What’s more, the U-shaped pattern of the PTA ratio and PFS was respectively validated in the two validation sets.

**Discussion:**

A U-shaped association between the PTA ratio and PFS was found in patients with DLBCLs. The PTA ratio can be used as a biomarker, and may suggest abnormalities of both host nutritional aspect and systemic inflammation in DLBCL.

## Introduction

Diffuse large B-cell lymphoma (DLBCL) is an entity of clinically and biologically heterogeneous non-Hodgkin lymphoma (NHL), accounting for approximately 30% of NHL. Although survival rates of DLBCL patients can achieve up to 60%–70% after standard therapy, over 50% of patients will experience relapse and lead to the short survival (1). In order to early identify the refractory and/or relapsed disease, useful biomarkers are critical in clinical practice. Among these, activated B-cell-like (ABC) subtype, double expressor lymphoma (DEL, double expression of the MYC and BCL2 oncogenes), and double- or triple-hit lymphomas (DH/THLs, genetic rearrangements of MYC and BCL2 and/or BCL6) have been proven to obtain adverse clinical outcomes (2). Recently, four, five, and seven genetic subtypes were respectively reported by Schmitz (3), Chapuy (4), and George (5) and colleagues proposed as a novel method for the precision medicine. Although there has been significant progression in the discovery of molecular biomarkers based on gene/protein expression profiling and mutational analyses, clinical parameters are still the mainstay for DLBCL classifications. For example, the international prognostic index (IPI) based on clinical parameters (6, 7) including age, disease stage, extranodal involvement, poor performance status, elevated lactate dehydrogenase, and its modified tools such as NCCN-IPI (7) and revised IPI (R-IPI) (6) are routinely used to estimate patients’ prognostication in the rituximab era. In addition, recent studies had demonstrated that other clinical parameters, like lymphocyte-to-monocyte ratio, serum albumin, and C-reactive protein, have the robust prognostic values in DLBCL patients (8–10).

Notably, a decreased platelet count, also termed thrombocytopenia, was regarded as an adverse prognostic factor in DLBCL (11). The causes of thrombocytopenia are multifactorial. IL-6 produced by lymphoma cells might contribute to thrombocytopenia. In addition, the bone marrow involvement of lymphoma cells was also reported as the cause of thrombocytopenia (12). At the same time, an elevated platelet count is an indicator of cancer (13). Platelets are considered as a part of the tumor microenvironment (14, 15). Interactions between platelets and tumor cells play an important role in cancer cell proliferation and metastasis. Platelets help tumors cells attach to endothelial cells for distant metastasis (15). In addition, tumor cells induce platelets to release growth factors in order to escape immune surveillance. Thus, an elevated platelet count is considered as an inflammatory biomarker and reflects the inflammatory response in lymphoma patients. Furthermore, consumptive character of malignant lymphomas has significant impact on nutritional status and host immune system (16).

Recently, a combinational index of platelet counts and albumin concentrations had been proven as an independent prognostic predictor in several diseases such as nasopharyngeal carcinoma (17), pancreatic adenocarcinoma (18), esophageal cancer (19), and osteosarcoma (20). However, whether the platelet-to-albumin (PTA) ratio has somewhat prognostic indication in DLBCL is still not investigated. Therefore, we analyzed the prognostic value of the PTA ratio in a large cohort of DLBCL patients as a training set and validated in two independent cohorts of patients.

## Materials and methods

We retrospectively recruited 749 patients in the First Affiliated Hospital, Zhejiang University School of Medicine (FAHZU), and 110 patients from Lishui Municipal Central Hospital (LMCH) from 2015 to 2020. All patients in this study were confirmed by pathological diagnoses. The inclusion criteria were as follows: patients’ serum albumin and platelet count were routinely tested at the time of disease diagnoses, and patients had no previous malignancy or secondary tumor. Patients with severe hepatic or renal insufficiency, HIV infection, transformed indolent lymphoma, and post-transplant DLBCL were excluded. Patients with pregnancy, complicated by another cancer, and disagreed follow-up were also not included for this study. Patients received four to six cycles of R-CHOP chemotherapy. The treatment regimens were as follows: rituximab 375 mg/m^2^ on day 0; cyclophosphamide 750 mg/m^2^, doxorubicin 50 mg/m^2^, and vincristine 1.4 mg/m^2^ on day 1; and prednisone 60 mg/m^2^ orally on days 1–5. The Ann Arbor Staging System, treatment response, and disease progression were investigated by clinical and laboratory examinations, computed tomography (CT) scans and/or positron emission tomography-CT, and bone marrow biopsy. Response was defined according to the Revised Response Criteria for Malignant Lymphoma (19). Pretreatment clinical and laboratory information was retrospectively collected from the medical records as follows: age, sex, Eastern Cooperative Oncology Group (ECOG) performance status score, extranodal involvement, lactate dehydrogenase (LDH), platelet counts, serum albumin concentrations, Ann arbor stage, COO classification, double expressor lymphoma (DEL), and treatment response (**Table 1**). All of the subjects were well-informed about the study and provided written informed consent to participate in the study. The study was approved by the Institutional Review Boards of the First Affiliated Hospital, Zhejiang University School of Medicine.

**Table 1:** Clinical characteristics of patients in this study.

Variables	Number (%)	Median(IQR)
All patients	859 (100)	
PTA ratio		4.99 (3.75 6.33)
Platelet cell counts, 10^9/L		213 (157, 265)
Albumin, g/L		42.9 (38, 46.5)
Sex, Male	469 (54.6)	
Female	390 (45.4)	
Age, years		60 (51 , 68)
LDH, U/L		257 (201 , 393)
ECOG-PS >2	251 (29.22)	
Ann Arbor Stage III-IV	566 (66.12 )	
Extranodal disease	482 (56.31 )	
IPI		
Low	205 (23.86)	
Low-intermediate	202 (23.52 )	
High-intermediate	226 (26.31 )	
High	226 (26.31 )	
Double-expressor lymphoma	311 (36.2)	
Non-GCB subtype	571 (66.47)	
Treatment response		
CR	623 (72.53 )	
PD	135 (15.72 )	
PR	59 (6.87)	
SD	42 (4.89)	

PTA, platelet-to-albumin; IQR, interquartile range; ECOG-PS, eastern cooperative oncology group performance status; LDH, lactate dehydrogenase; IPI, International Prognostic Index. Extranodal involvement: the bone marrow, CNS, liver/GI tract, spleen, lung and other sites.

### Immunohistochemical analyses

Formalin-fixed paraffin-embedded samples were used to detect the proteins expression. Sections were stained with antibodies of CD10 (Zhong Shan-Golden Bridge Biological Technology Co., Ltd), BCL6 (Zhong Shan-Golden Bridge Biological Technology Co., Ltd), and MUM1 (Shanghai Changdao Biological Technology Co., Ltd). Staining of the tumor cells 30% or more was considered positive (21). Immunostaining was performed using the Envision System with diaminobenzidine (Dako, Glostrup, Denmark). Cell-of-origin (COO) classification was determined by the Hans algorithms (21). Cases with more than 40% positive cells of MYC and 50% of BCL2 were identified as DEL.

### Platelet-to-albumin ratio measurements

The PTA ratio was calculated by utilizing the formula [PTA= platelet counts (10^9^/L)/serum albumin (g/dl)] derived from the complete blood counts and biochemistry tests at the time of disease diagnosis.

### Statistical analysis

In the training set, 600 patients were used to explore the prognostic impact of the PTA ratio on progression-free survival (PFS). PFS was used as the primary endpoint. PFS was defined as time from date of diagnosis until removal from the study due to non-complete remission, relapse, or death. OS was defined as time from the date of diagnosis until death due to any cause or the last follow-up. Penalized smoothing spline (PS) Cox regression models were used to explore the non-linear relationship between the PTA ratio and OS and PFS, respectively. Two optimal cutoff values were estimated by the “CutpointsOEHR” package (22) and refined patients into three subgroups. The prognostic impact of the three subgroups was investigated by the log-rank test in the Kaplan–Meier survival model. Stratified analysis was also performed to assess the impact of confounding variables. Using 24% and 51% of the 3-year PFS rates in the low and intermediate groups, we estimated the sample sizes for the validation set by a log-rank test. A sample size from 100 to 150 patients achieves a power from 83% to 93% to detect a difference of 3-year PFS rates between 24% and 51% at a 0.05 significance level. Thus, 149 patients in our hospital were selected as the internal validation set, and 110 patients from a different hospital were used as the external validation set. The proportional-hazards assumption was checked for each variable before fitting Cox models. Univariate and multivariate analyses with a Cox proportional hazard model were performed to assess significant predictors. The median, interquartile range, and frequency counts were used to summarize the distribution of clinical parameters. Fisher’s exact test and non-parameter T-test were respectively used to test the categorical and continuous variables. All statistical analyses were conducted with R statistic packages, version 3.5.1 (www.r-project.org). The two-sided level of significance was set at p-value < 0.05.

## Results

### Patient characteristics

**Figure 1:**
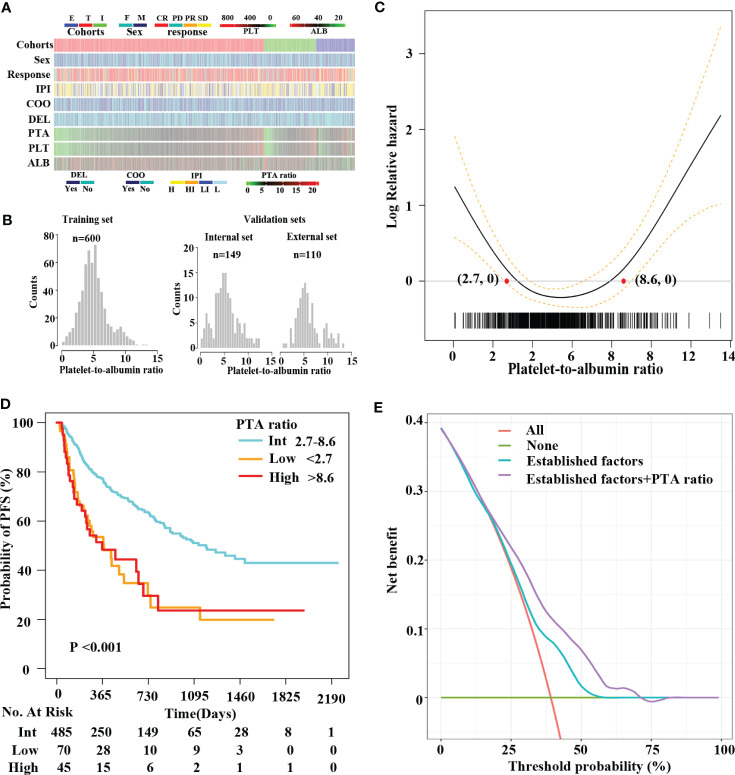
A U-shape relationship between the PTA ratio and PFS in DLBCL patients. Clinical features of patients in this study **(A)**. Distribution of the PTA ratio in the training (T) set and the internal (I) and external (E) validation sets **(B)**. IPI includes high-risk (H), high-intermediate-risk (HI), low-intermediate-risk (LI), and low-risk (L) groups. Penalized smoothing splines model demonstrated a U-shape relation between the PTA ratio and PFS **(C)**. Two optimal cutoff values of the PTA ratio [2.7, 8.6] are illustrated in red dots **(C)** and refined patients into three subgroups **(D)**: low, intermediate, and high groups, respectively. Kaplan–Meier curves revealed patients in the low and high groups had shorter PFS compared to those in the intermediate group **(D)**. Decision curve analysis showing the clinical utility of the well-established predictors (combining sex, COO classification, DEL, and IPI) with and without the PTA ratio in the Cox regression analysis **(E)**. The red straight line represents the net benefit of treating all patients without the PTA ratio, assuming that all patients would survive. The green straight line represents the net benefit of treating all patients similarly, assuming that all would die. The cyan line represents the net benefit of treating patients according to the combination of the well-established factors including sex, COO classification, DEL, and IPI. The purple line represents the net benefit of treating patients according to the well-established predictors combining with the PTA ratio.

In total, 859 patients were newly diagnosed with diffuse large B-cell lymphoma (DLBCL), with 469 (54.6%) men and 390 (45.4%) women in this study (**Table 1**, **Figure 1A**). The median age was 60 (range, 18–88). Median follow-up for DLBCL patients was 534 days (interquartile range, 206–757), with 678 (78.9%) and 513 (59.7%) patients being still alive and free of disease progression during the final analysis. The characteristics of DLBCL patients in the training and internal validation sets are as depicted in **Table 2**. There was no difference in clinical characteristics including sex, age, platelet counts, serum albumin concentrations, PTA ratio, international prognostic index (IPI), cell-of-origin (COO) classification, and double expressor lymphoma (DEL) subtypes between the training and internal validation sets. Of note, the median overall survival (OS) was not yet reached for patients in the training set and 809 days in the internal validation set; the median PFS was 984 and 657 days for the training and internal validation sets, respectively. The 3-year OS rates were 66.7% (CI, 61.1%–72.8%) and 43.5% (CI, 29.3%–64.5%) for the training and internal validation sets, respectively. The 3-year PFS rates were 46.8% (CI, 41.5%–52.6%) and 41.2% (CI, 29.6%–57.3%) for the training and internal validation sets, respectively (**Supplementary Figure S1**).

**Table 2:** Clinical characteristics of patients.

Variables	Training set	Internal validation set	External validation set	Pt values	Pi values	Pe values
Number	600	149	110			
PTA ratio, median[IQR]	4.89[3.68,6.13]	5.05[3.63,6.69]	5.39[4.36,7.08]	0.002	0.390	<0.001
PLT (10^9/L, median[IQR])	212.00[157.00,262.00]	214.00[146.00,278.00]	215.00[161.25,257.75]	0.877	0.870	0.613
ALB (g/L, median[IQR])	43.90[39.40,47.00]	43.10[36.80,45.60]	37.30[32.92,40.68]	<0.001	0.058	<0.001
Sex, male, n(%)	321(53.5)	82(55.0)	66(60.0)	0.457	0.783	0.213
Age, years	59.00[51.00,67.00]	62.00[50.00,68.00]	65.00[53.00,73.00]	<0.001	0.347	<0.001
LDH (U/L, median[IQR])	263.00 [208.00,397.00]	257.00 [195.00,423.00]	225.00[169.00,339.00]	0.010	0.939	0.003
ECOG-PS>2, n(%)	162(27.0)	37(24.8)	52(47.3)	<0.001	0.679	<0.001
Ann Arbor Stage III-IV, n(%)	376(63.0)	105(70.5)	85(77.3)	0.006	0.104	0.004
Extranodal disease, n(%)	319(53.3)	78(52.7)	85(77.3)	<0.001	0.927	<0.001
IPI, n(%)				<0.001	0.38	<0.001
Low	157(26.2)	34(22.8)	14(12.7)			
Low-intermediate	145(24.2)	31(20.8)	26(23.6)			
High-intermediate	156(26.0)	49(32.9)	21(19.1)			
High	142(23.7)	35(23.5)	49(44.5)			
Double-expressor lymphoma, n(%)	218(36.3)	63(42.3)	NA	0.045	0.187	NA
Non-GCB subtype, n(%)	390(65.0)	104(69.8)	77(70.0)	0.389	0.289	0.327
Treatment response, n(%)				<0.001	0.076	0.007
CR	439(73.2)	108(72.5)	76(69.1)			
PD	94(15.7)	30(20.1)	11(10.0)			
PR	40(6.7)	10(6.7)	9(8.2)			
SD	27(4.5)	1(0.7)	14(12.7)			

PTA, platelet-to-albumin; IQR, interquartile range; ECOG-PS, eastern cooperative oncology group performance status; LDH, lactate dehydrogenase; IPI, International Prognostic Index. Extranodal involvement: the bone marrow, CNS, liver/GI tract, spleen, lung and other sites. CR, complete remission; PD, progressive disease; PR, partial response; SD, stable disease. Pt values indicate differences among three groups. Pi and Pe values are derived from the comparison between the training set and the internal as well as external validation sets.

### Prognostic impact of the PTA ratio as a continuous variable in the training set

The PTA ratio showed a slightly skewed right distribution in the training and validation sets (**Figure 1B**, **Supplementary Figure S2**). First, we evaluate the relationship between the PTA ratio and PFS using the penalized smoothing splines (PS) models in Cox regression analyses. As a result, the estimated survival curves of the PTA ratio exhibited a U-shaped hazard ratio for PFS, with a nadir in the intermediate values of the PTA ratio, the sharp fall in the low values, and then increase again in the high values (**Figure 1C**). Additionally, when we considered the well-established prognostic factors as confounders, the non-linear pattern between the PTA ratio and PFS was still observed in PS models after adjusting for sex, IPI, DEL subtypes, and COO classification (**Supplementary Figure S3A**). Similarly, the U-shaped pattern between the PTA ratio and OS was found in univariate and multivariate analyses, respectively (**Supplementary Figures S3B, C**).

### Prognostic impact of the PTA ratio as a three-categorical variable in the training set

We refined 600 patients into three subgroups based on two optimal cutoff values of the PTA ratio (**Figure 1C**), Accordingly, 70 (11.7%), 485 (80.8%), and 45 (7.5%) patients were classified as low, intermediate, and high group, respectively; their 3-year PFS and OS rates were 34.2% and 50.4%, 50.9% and 71.0%, and 24.3% and 51.0% for the low, intermediate, and high group, respectively (**Figure 1D**, **Supplementary Figure S4**). Clinical characteristics of patients in three groups are summarized in **Supplementary Table S1**. Notably, high and low groups had somewhat similar clinical characteristics. Specifically, both low and high groups were more common in men and had Ann Arbor Stage III–IV, ECOG performance score > 2, and disease progression, lower concentrations of serum albumin, elevated LDH levels, and high IPI scores than the intermediate group. Additionally, patients in the low group had lower platelet counts and lower complete remission rates. In contrast, patients in the high group had higher platelet counts. There was no statistically significant correlation between the PTA ratio and other variables including age, COO classification, and DEL subtypes.

In this study, several factors were associated with poor outcomes, including sex, IPI, and DEL subtype in univariate analyses for OS or PFS (**Supplementary Table S** **2**). Furthermore, the PTA ratio as a three-categorical variable was not significantly confounded by the well-established predictors including sex, COO classification, DEL subtype, and IPI (**Supplementary Tables S3, S4**). In multivariate analyses, the PTA ratio as a three-categorical variable, sex, IPI, and DEL subtypes maintained a significant association with poor outcomes (**Table 3**, **Supplementary Table S5**).

**Table 3:** Multivariable analyses for PFS in this study.

Variables	The training set		The internal validation set		The external validation set	
	P value	HR(95% CI)	P value	HR(95% CI)	P value	HR(95% CI)
PTA ratio						
Low vs. Intermediate	0.010	1.565(1.112,2.201)	0.005	2.396(1.301,4.41)	0.023	3.068(1.169,8.05)
High vs. Intermediate	0.009	1.763(1.155,2.690)	0.005	2.98(1.395,6.363)	0.002	3.127(1.503,6.505)
Male vs. Female	0.014	1.390(1.069,1.808)	0.036	1.824(1.039,3.201)	0.246	1.429(0.782,2.611)
IPI						
Low-intermediate vs. Low	<0.001	2.585(1.605,4.163)	0.021	6.060(1.316,27.898)	0.107	5.529(0.689,44.342)
High-intermediate vs. Low	<0.001	3.400(2.166,5.337)	0.012	6.587(1.513,28.665)	0.041	8.749(1.097,69.781)
High vs. Low	<0.001	5.524(3.527,8.651)	0.001	13.003(3.004,56.282)	0.017	11.575(1.555,86.165)
GCB VS. Non-GCB	0.508	1.099(0.831,1.451)	0.124	1.684(0.867,3.271)	0.120	1.736(0.865,3.483)
DEL(Yes vs.NO)	0.068	1.279(0.982,1.665)	0.57	1.176(0.672,2.06)	NA	NA

PTA, platelet-to-albumin; IPI, International Prognostic Index; DEL, double expressor lymphoma; COO, cell-of-origin.

On decision curves analysis, the addition of the PTA ratio to the multivariate analysis models after adjusting for sex, IPI, COO classification, and DEL subtype resulted in significant net benefits for both PFS and OS (**Figure 1E**, **Supplementary Figure S5**). On the multivariate model after adjusting for the well-established predictors including sex, COO classification, DEL, and IPI, net benefits were obtained for OS and PFS between threshold probabilities of 10%–30% and 10%–60% (**Figure 1E**, **Supplementary Figure S5**), respectively. In contrast, after adjusting for the above well-established predictors and the PTA ratio, net benefits for OS and PFS increased between threshold probabilities of 10%–60% and 10%–75%, respectively. These results strongly indicated that the PTA ratio had an additional value of predicting survival for patients with DLBCL.

### The U-shaped pattern between the PTA ratio and PFS was confirmed in the internal validation set

**Figure 2:**
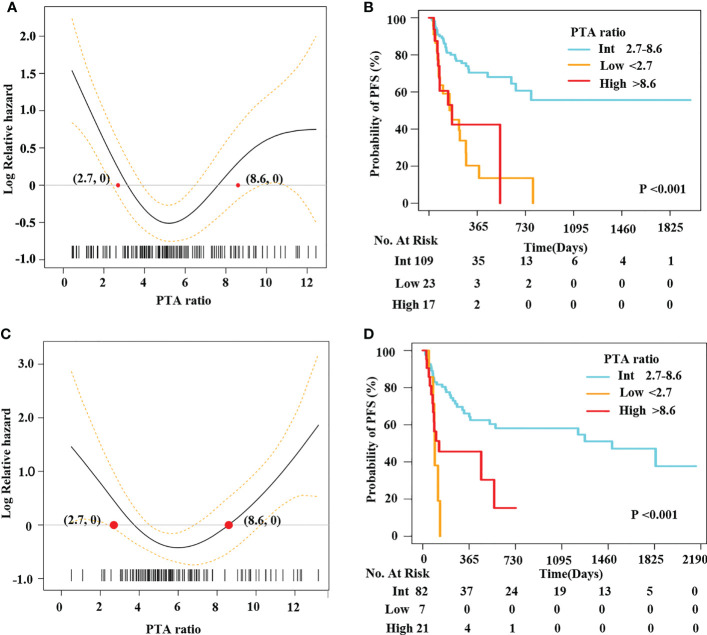
A U-shape relationship between the PTA ratio and PFS was validated in the internal and external validation sets. Penalized smoothing splines model demonstrated a U-shape relation between the PTA ratio and PFS in 149 patients from our hospital **(A)** and an independent cohort of LMCH patients **(C)**. Two optimal cutoff values of the PTA ratio [2.7, 8.6] classified patients into three subgroups: low, intermediate, and high group from our hospital **(B)** and LMCH **(D)**.

Herein, 149 patients were randomly selected in our hospital as the internal validation set. We used the same statistical methods to evaluate the relationship between the PTA ratio and outcomes. First, concerning the PTA ratio as a continuous variable, PS models demonstrated U-shaped patterns between the PTA ratio and PFS and OS (**Figure 2A**, **Supplementary Figure S6A**). Of note, using the two cutoff values from the training set, we identified 17 (11.4%) patients as the high group and 23 (15.4%) patients as the low group, respectively. Clinical features of these patients are shown in **Supplementary Table S6**. The high and low groups had the adverse outcomes with respect to PFS and OS in univariate and multivariate analyses (**Figure 2B**, **Supplementary Figure S7**, **Table 3**, **Supplementary Tables S7, S8**). Third, after adjusting for the well-established predictors including sex, COO classification, DEL, and IPI, the PTA ratio as a continuous variable was still associated with survival in multivariate models (**Supplementary Figures S6B, C**). Decision curves analysis also illustrated that the PTA ratio had an additional value to predict survival in the internal validation set from the same hospital (**Supplementary Figure S8**).

### The U-shaped pattern between the PTA ratio and PFS was further confirmed in the external validation set

We enrolled 110 patients from LMCH as an external validation set. Clinical features of patients are summarized in **Table 2**. We found the PTA ratio was higher in patients from LMCH than those from FAHZU, partly due to the lower concentrations of serum albumin in LMCH patients. These patients were older, more common in Ann Arbor Stage III–IV, ECOG performance score > 2, and high IPI scores than patients in the training set. The 3-year OS and PFS rates were 72.9% (CI, 63.4%–86.4%) and 48.7% (CI, 38.8%–61.0%) for these patients, respectively (**Supplementary Figure S1**). The U-shaped relations between the PTA ratio and PFS and OS were still evident in the univariate analyses by PS models (**Figure 2C**, **Supplementary Figure S9A**). Additionally, the U-shaped pattern between PFS and the PTA ratio was still found in the context of the well-established factors like sex, IPI, and COO classification (**Supplementary Figure S9B**). When patients were classified into three subgroups using the cutoff value of the PTA ratio [2.7, 8.6], patients with the low PTA ratio had the adverse PFS and OS in univariate analyses (**Figure 2D**, **Supplementary Figure S10**, **Supplementary Table S9**). Clinical features of three subgroups are shown in **Supplementary Table S10**. Notably, the non-linear association between the PTA ratio as a three-categorical variable remained for PFS in the multivariate analyses, but the statistical significance disappeared for OS (**Supplementary Figure S9B, C**, **Table 3**, **Supplementary Table S11**).

## Discussion

Diffuse large B-cell lymphoma (DLBCL) represents a group of substantial heterogeneous diseases in terms of their biological insights and clinical features. By now, morphological, immunophenotypic, molecular, and genetic biomarkers have allowed to refine patients into distinct subgroups. However, some cases still cannot be classified and collectively termed DLBCL not otherwise specified. Thus, the useful biomarker is still required in clinical practice. Prognostic parameters are of great importance to identify high-risk patients who might benefit from novel therapeutic agents. To date, molecular markers derived from gene expression profiling like COO classification and next-generation sequencing such as genetic subtypes are critical to refine patients into distinct risk subgroups. However, some questions are worthy of noting before implementing these novel biomarkers. First, the detection of molecular markers often limited the adoption in some developing countries because it is usually expensive and required for high-technical approaches. In contrast, clinical parameters like IPI are regarded as a useful tool for disease classification and outcome prediction. Furthermore, considering cost-effective services, utilities of the composition of two clinical parameters to construct a parameter would be a better method to explore a practical predictor. Therefore, we evaluate whether the PTA ratio could be used as a parameter to predict outcomes of DLBCL patients.

With a growing body of evidence on the role of host immunity and nutrition in disease progression, the prognostic value of related biomarkers has been investigated in DLBCL. Serum albumin is a surrogate for nutritional aspect and disease severity. Malnutrition is associated with adverse outcome in DLBCL patients (9). In present studies, platelets have a bi-directional function in the process of cancer progression (15). At the same time, the prognostic significance of the platelet counts has not been determined. Some studies suggested that decreased platelet counts were an unfavorable prognostic predictor (11–13, 16). In contrast, others proposed decreased platelet counts as a favorable biomarker (14). The underlying reason for the discrepancy might be that the relationship between the platelet counts and prognoses might be not necessarily linear but might be U-shaped. In fact, platelet is an inflammatory biomarker. Thus, elevated or decreased platelet counts, reflecting dysfunction of immune system, might contribute to the adverse outcome. The PTA ratio, consisting of albumin and platelets, is considered as a marker to reflect host nutrition status and systemic inflammation. Therefore, the PTA ratio is of great significance for the research of the relationship between systemic inflammation response and progression of disease in DLBCL patients. First, the PTA ratio presents with a non-normal distribution as shown in **Figure 1**. From the non-normal distribution, we hypothesized that changes in the PTA ratio might obtain the non-linear impact of clinical outcomes. Therefore, we conducted PS models to investigate the non-linear effect of the PTA ratio on PFS. As a result, we found a U-shaped relationship between the PTA ratio and PFS (**Figure 1C**). At the same time, these U-shaped effects of PTA ratio on outcomes were evident when the PTA ratio was divided into three subgroups. Specifically, DLBCL patients with the low and high levels of the PTA ratio had the shorter survivals compared to those in the intermediate values (**Figure 1D**). Second, even if we considered the well-established predictors like IPI, sex, COO classification, and DEL as potential confounders, we found no interaction between the PTA ratio and these well-established factors. Finally, after adjusting for IPI, sex, COO classification, and DEL, the PTA ratio as a continuous or three-categorical variable was respectively an independent predictor in the training set and the two independent validation sets. Of note, comparing with the well-established factors, changes in the PTA ratio contributed to the additional prognostic significance in DLBCL patients. Therefore, we demonstrated that the PTA ratio could be used as a predictor for DLBCL patients. We hope that the U-shaped relationship between PTA ratio and PFS may be used to guide “personalized immunotherapy” for lymphoma.

There are still some limitations in this study. First, we did not examine genetic mutation subtypes in our patients; thus, we could not exclude these molecular biomarkers, which will confound the prognostic value of the PTA ratio in lymphoma patients. Second, this is a retrospective study. A prospective cohort in the multicenters is mandatory to examine our results. Therefore, caution in the application of our findings is still warranted.

## Conclusion

In this study, we present a U-shaped relationship between the PTA ratio and PFS in patients with DLBCL. The PTA ratio can be used as a surrogate for disease severity and may suggest abnormalities of both host nutritional aspect and systemic inflammation in DLBCL patients.

## Data availability statement

The original contributions presented in the study are included in the article/**Supplementary Material**. Further inquiries can be directed to the corresponding authors.

## Ethics statement

The studies involving human participants were reviewed and approved by the Institutional Review Boards of the First Affiliated Hospital, Zhejiang University School of Medicine. The patients/participants provided their written informed consent to participate in this study.

## Author contributions

JW, FY, and JJ: study design, data interpretation, writing, and approval of the final manuscript. JW, LM, BC, XH, JZ, HZ, WX, LL, HT, and JJ: data collection. JJ and LL take responsibility for the integrity of the data. All authors contributed to the article and approved the submitted version.
