# Supplementary material for: Development and validation of platelet-to-albumin ratio as a clinical predictor for diffuse large B-cell lymphoma
Source: Front Oncol. 2023 Jun 8;13:1138284. doi: 10.3389/fonc.2023.1138284 (PMC10285288; doi:10.3389/fonc.2023.1138284)
Supplement: Supplementary file 1 [file DataSheet_1.docx]

**A redacted protocol for this study:**

**Selection of patients, including both eligibility and ineligibility criteria**

All patients in this study were confirmed by pathological diagnoses. The inclusion criteria were as follows: patients were routinely tested serum albumin and platelet count at the time of disease diagnoses, and patients had no previous malignancy or secondary tumor. Patients with severe hepatic or renal insufficiency, HIV infection, transformed indolent lymphoma, post-transplant DLBCL were excluded.

**Schema and treatment plan, including administration schedule**

Patients received 4 to 6 cycles of R-CHOP chemotherapy. The treatment regimens were as follows: rituximab 375 mg/m^2^ on Day 0, cyclophosphamide 750 mg/m^2^, doxorubicin 50 mg/m^2^, and vincristine 1.4 mg/m^2^ on Day 1, and prednisone 60 mg/m^2^ orally on Days 1-5.

**Rules for dose modification**
Patients with pregnancy, complicated by another cancer, and disagreed to follow-up were also not included for this study.
**Measurement of treatment effect including response criteria, definitions of response and survival, and methods of measurement**

The Ann Arbor Staging System, treatment response, and disease progression were investigated by clinical and laboratory examinations, computed tomography (CT) scans and/or positron emission tomography-CT, and bone marrow biopsy. Response was defined according to the Revised Response Criteria for Malignant Lymphoma^19^.

**Reasons for early cessation of trial therapy**

Patients disagreed to follow-up were also not included for this study. **Objectives and entire statistical section (including endpoints)**

Pretreatment clinical and laboratory information was retrospectively collected from the medical records as follows: age, sex, eastern cooperative oncology group (ECOG) performance status score, extranodal involvement, lactate dehydrogenase (LDH), platelet counts and serum albumin concentrations. PFS was defined as time from date of diagnosis until removal from study due to non-complete remission, relapse, or death. OS was defined as time from the date of diagnosis until death due to any cause or the last follow-up. Penalized smoothing spline (PS) Cox regression models were used to explore the non-linear relationship between the PTA ratio and OS as well as PFS, respectively.

Table -S1.Clinical characteristics of patients by the PTA ratio as a categorical variable in the training set

| Variables | Total patients | Intermediate group(IG) | Low group(LG) | High group(HG) | P values | | | |
| --- | --- | --- | --- | --- | --- | --- | --- | --- |
|  |  |  |  |  | Total | IG vs. LG | IG vs. HG | LG vs HG |
| Number | 600 | 485 | 70 | 45 |  |  |  |  |
| PTA ratio, median[IQR] | 4.89[3.68;6.13] | 4.99 [4.02;5.94] | 2.05 [1.34;2.43] | 9.80 [9.36;10.5] | <0.001 | <0.001 | <0.001 | <0.001 |
| PLT (10^9/L, median[IQR]) | 212[157;262] | 216 [175;257] | 82.0 [54.5;108] | 398 [352;445] | <0.001 | <0.001 | <0.001 | <0.001 |
| ALB (g/L, median[IQR]) | 43.9[39.4;47.0] | 44.2 [40.0;47.3] | 43.2 [36.6;46.5] | 39.6 [34.9;44.7] | <0.001 | 0.105 | <0.001 | 0.105 |
| Sex, male, n(%) | 321(53.5) | 248 (51.1%) | 45 (64.3%) | 28 (62.2%) | 0.057 | 0.160 | 0.307 | 0.979 |
| Age, years | 59.0[51.0;67.0] | 59.0 [51.0;67.0] | 62.0 [53.0;67.0] | 62.0 [49.0;67.0] | 0.82 | 0.928 | 0.928 | 0.928 |
| LDH (U/L, median[IQR]) | 263[208;397] | 253 [204;349] | 385 [242;727] | 304 [223;464] | <0.001 | <0.001 | 0.059 | 0.136 |
| ECOG-PS>2, n(%) | 162(27.0) | 117 (24.1%) | 26 (37.1%) | 19 (42.2%) | 0.004 | 0.044 | 0.039 | 0.727 |
| Ann Arbor Stage III-IV, n(%) | 376(63.0) | 291 (60.4%) | 51 (72.9%) | 34 (75.6%) | 0.025 | 0.098 | 0.098 | 0.917 |
| Extranodal disease, n(%) | 319(53.3) | 251 (52.0%) | 42 (60.0%) | 26 (57.8%) | 0.374 | 0.775 | 0.832 | 0.966 |
| IPI, n(%) |  |  |  |  | 0.002 | 0.014 | 0.016 | 0.792 |
| Low | 157(26.2) | 140 (28.9%) | 11 (15.7%) | 6 (13.3%) |  |  |  |  |
| Low-intermediate | 145(24.2) | 121 (24.9%) | 13 (18.6%) | 11 (24.4%) |  |  |  |  |
| High-intermediate | 156(26.0) | 126 (26.0%) | 20 (28.6%) | 10 (22.2%) |  |  |  |  |
| High | 142(23.7) | 98 (20.2%) | 26 (37.1%) | 18 (40.0%) |  |  |  |  |
| Double-expressor lymphoma,n(%) | 218(36.3) | 176 (36.3%) | 24 (34.3%) | 18 (40.0%) | 0.823 | 0.847 | 0.847 | 0.847 |
| Non-GCB subtype, n(%) | 390(65.0) | 319 (65.8%) | 43 (61.4%) | 28 (62.2%) | 0.714 | 1 | 1 | 1 |
| Treatment response, n(%) | |  |  |  | 0.044 | 0.036 | 0.563 | 0.878 |
| CR | 439(73.2) | 368 (75.9%) | 41 (58.6%) | 30 (66.7%) |  |  |  |  |
| PD | 94(15.7) | 65 (13.4%) | 19 (27.1%) | 10 (22.2%) |  |  |  |  |
| PR | 40(6.67) | 31 (6.39%) | 6 (8.57%) | 3 (6.67%) |  |  |  |  |
| SD | 27(4.50) | 21 (4.33%) | 4 (5.71%) | 2 (4.44%) |  |  |  |  |

PTA; platelet-to-albumin, IQR; interquartile range, ECOG-PS; eastern cooperative oncology group performance status, LDH; lactate dehydrogenase, IPI; International Prognostic Index, Extranodal involvement: the bone marrow, CNS, liver/GI tract, spleen, lung and other sites.

Table S2. Univariate analyses of the PTA ratio as a categorical variable for survivals in the training set

|  | Overall survival | | Progression-free survival | |
| --- | --- | --- | --- | --- |
| Variables | P value | HR (95% CI) | P value | HR (95% CI) |
| PTA ratio | |  |  |  |
| **Low vs. Intermediate** | <0.001 | 2.644(1.734,4.032) | <0.001 | 1.827(1.306,2.557) |
| **High vs. Intermediate** | <0.001 | 2.782(1.597,4.849) | <0.001 | 2.385(1.575,3.611) |
| **Male vs. Female** | 0.124 | 1.324(0.926,1.892) | 0.012 | 1.393(1.075,1.805) |
| **IPI** |  |  |  |  |
| **Low-intermediate vs. Low** | 0.006 | 2.572(1.308,5.056) | <0.001 | 2.743(1.709,4.403) |
| **High-intermediate vs. Low** | <0.001 | 3.844(2.044,7.23) | <0.001 | 3.596(2.296,5.632) |
| **High vs. Low** | <0.001 | 6.299(3.407,11.648) | <0.001 | 6.086(3.91,9.474) |
| **GCB vs. Non-GCB** | 0.77 | 1.057(0.728,1.535) | 0.238 | 1.18(0.896,1.552) |
| **DEL(Yes vs.NO)** | 0.013 | 1.568(1.098,2.238) | 0.023 | 1.353(1.043,1.756) |

PTA; platelet-to-albumin, IPI; International Prognostic Index, DEL, double expressor lymphoma, COO; cell-of-origin

Table S3. Interaction analyses of FPS by the PTA ratio as a categorical variable in the training set

| Variables | Low (n) | Intermediate (n) | High | Low vs. Intermediate group | |  | High vs. Intermediate group | |  |
| --- | --- | --- | --- | --- | --- | --- | --- | --- | --- |
|  |  |  |  | P value | HR(95%CI) | Pi value | P value | HR(95%CI) | Pi value |
| PTA classifications | 84 | 470 | 46 | <0.001 | 1.827(1.306,2.557) |  | <0.001 | 2.385(1.575,3.611) | |
| Sex | 84 | 470 | 46 | 0.001 | 1.766(1.26,2.475) | 0.184 | <0.001 | 2.277(1.5,3.457) | 0.584 |
| Female | 29 | 233 | 17 | 0.362 | 1.315(0.73,2.369) | | 0.003 | 2.862(1.427,5.74) | |
| Male | 55 | 237 | 29 | 0.001 | 2.085(1.373,3.166) | | 0.006 | 2.087(1.24,3.512) | |
| CCO classification | 84 | 470 | 46 | <0.001 | 1.833(1.31,2.567) | 0.678 | <0.001 | 2.35(1.551,3.561) | 0.754 |
| GCB | 31 | 161 | 18 | 0.019 | 2.006(1.122,3.586) | | 0.006 | 2.619(1.321,5.195) | |
| Non-GCB | 53 | 309 | 28 | 0.008 | 1.756(1.162,2.654) | | 0.003 | 2.217(1.313,3.745) | |
| DEL | 84 | 470 | 46 | <0.001 | 1.893(1.35,2.656) | 0.173 | <0.001 | 2.321(1.53,3.519) | 0.058 |
| NO | 58 | 297 | 27 | 0.043 | 1.575(1.013,2.447) | | <0.001 | 3.296(1.962,5.539) | |
| Yes | 26 | 173 | 19 | <0.001 | 2.65(1.566,4.484) | | 0.259 | 1.493(0.744,2.996) | |
| IPI risk groups | 84 | 470 | 46 | 0.012 | 1.545(1.098,2.174) | 0.586 | 0.002 | 1.949(1.279,2.97) | 0.292 |
| Low | 14 | 136 | 7 | 0.945 | 0.95(0.223,4.046) | | 0.065 | 3.947(0.916,16.999) | |
| Low-intermediate | 17 | 117 | 11 | 0.315 | 1.514(0.674,3.399) | | 0.425 | 1.464(0.574,3.73) | |
| High-intermediate | 24 | 122 | 10 | 0.02 | 2.023(1.118,3.661) | | 0.083 | 2.118(0.906,4.951) | |
| High | 29 | 95 | 18 | 0.208 | 1.395(0.831,2.341) | | 0.035 | 1.934(1.046,3.574) | |

P values resulted from variables in high- or low-group comparing with those in intermediate-group. Pi values resulted from their interaction analyses. “P value” of stratification analysis less than 0.05 for the adjusted HR, which is in line with the P value for crude HR, indicates this factor was not a significant confounder.

Table S4. Interaction analyses of OS by the PTA ratio as a three categorical variable in the training set

| Variables | Low (n) | Intermediate (n) | High | Low vs. Intermediate group | |  | High vs. Intermediate group | |  |
| --- | --- | --- | --- | --- | --- | --- | --- | --- | --- |
|  |  |  |  | P value | HR(95%CI) | Pi value | P value | HR(95%CI) | Pi value |
| PTA classifications | 84 | 470 | 46 | <0.001 | 2.644(1.734,4.032) | | <0.001 | 2.782(1.597,4.849) | |
| Sex | 84 | 470 | 46 | <0.001 | 2.555(1.673,3.902) | 0.125 | 0.001 | 2.701(1.539,4.741) | 0.931 |
| Female | 29 | 233 | 17 | 0.150 | 1.702(0.825,3.512) | | 0.039 | 2.984(1.054,8.443) | |
| Male | 55 | 237 | 29 | <0.001 | 3.31(1.935,5.662) | | 0.003 | 2.757(1.406,5.406) | |
| CCO classification | 84 | 470 | 46 | <0.001 | 2.76(1.809,4.213) | 0.957 | 0.001 | 2.67(1.533,4.648) | 0.827 |
| GCB | 31 | 161 | 18 | 0.004 | 2.967(1.42,6.198) | | 0.025 | 2.761(1.134,6.723) | |
| Non-GCB | 53 | 309 | 28 | <0.001 | 2.666(1.591,4.468) | | 0.008 | 2.62(1.289,5.328) | |
| DEL | 84 | 470 | 46 | <0.001 | 2.724(1.782,4.164) | 0.232 | <0.001 | 2.691(1.542,4.694) | 0.188 |
| NO | 58 | 297 | 27 | <0.001 | 3.329(1.952,5.679) | | <0.001 | 3.843(1.851,7.979) | |
| Yes | 26 | 173 | 19 | <0.001 | 1.993(0.962,4.128) | | 0.183 | 1.799(0.759,4.263) | |
| IPI risk groups | 84 | 470 | 46 | <0.001 | 2.22(1.447,3.408) | 0.728 | 0.006 | 2.199(1.251,3.865) | 0.381 |
| Low | 14 | 136 | 7 | 0.987 | 0.983(0.125,7.707) | | 0.011 | 7.442(1.591,34.81) | |
| Low-intermediate | 17 | 117 | 11 | 0.496 | 1.461(0.49,4.353) | | 0.452 | 1.602(0.469,5.475) | |
| High-intermediate | 24 | 122 | 10 | 0.013 | 2.627(1.225,5.634) | | 0.77 | 1.24(0.293,5.252) | |
| High | 29 | 95 | 18 | 0.003 | 2.624(1.386,4.967) | | 0.019 | 2.649(1.17,5.997) | |

P values resulted from variables in low- and high-group respectively comparing with those in intermediate-group. Pi values resulted from their interaction analyses. “P value” of stratification analysis less than 0.05 for the adjusted HR, which is in line with the P value for crude HR, indicates this factor was not a significant confounder.

Table S5.Multivariate analyses of the PTA ratio as a categorical variable for OS in the training set

|  | Overall survival | |
| --- | --- | --- |
| Variables | P value | (95% CI) |
| PTA ratio | |  |
| **Low vs. Intermediate** | <0.001 | 2.308(1.502,3.546) |
| **High vs. Intermediate** | 0.008 | 2.147(1.216,3.789) |
| **Male vs. Female** | 0.204 | 1.268(0.879,1.829) |
| **IPI** |  |  |
| **Low-intermediate vs. Low** | 0.015 | 2.325(1.176,4.596) |
| **High-intermediate vs. Low** | <0.001 | 3.548(1.879,6.697) |
| **High vs. Low** | <0.001 | 5.32(2.855,9.914) |
| **GCB vs. Non-GCB** | 0.907 | 1.023(0.699,1.496) |
| **DEL(Yes vs.NO)** | 0.027 | 1.499(1.047,2.145) |

PTA; platelet-to-albumin, IPI; International Prognostic Index, DEL, double expressor lymphoma, COO; cell-of-origin

Table S6. Clinical features of 149 patients in the internal validation set

| Variables | Total patients | Intermediate group(IG) | Low group(LG) | High group(HG) | P values | | | |
| --- | --- | --- | --- | --- | --- | --- | --- | --- |
|  |  |  |  |  | Total | IGvs.LG | IGvs.HG | LGvsHG |
| Number | 149 | 109 | 23 | 17 |  |  |  |  |
| PTA ratio, median[IQR] | 5.05[3.63;6.69] | 5.13[4.18;6.12] | 1.40[0.96;1.96] | 10.0[9.44;11.5] | <0.001 | <0.001 | <0.001 | <0.001 |
| PLT (10^9/L, median[IQR]) | 214[146;278] | 220[180;263] | 63.0[46.0;73.0] | 392[337;440] | <0.001 | <0.001 | <0.001 | <0.001 |
| ALB (g/L, median[IQR]) | 43.1[36.8;45.6] | 43.3[39.0;45.6] | 43.9[40.2;50.9] | 36.8[34.4;41.4] | 0.004 | 0.149 | 0.006 | 0.006 |
| Sex, male, n(%) | 82(55.0) | 61(56.0) | 12(52.2) | 9(52.9) | 0.930 | 1 | 1 | 1 |
| Age, years | 62.0[50.0;68.0] | 61.0[50.0;67.0] | 63.0[58.0;70.5] | 54.0[45.0;64.0] | 0.140 | 0.200 | 0.260 | 0.200 |
| LDH (U/L, median[IQR]) | 257[195;423] | 239[189;347] | 501[322;1140] | 272[174;508] | <0.001 | <0.001 | 0.532 | 0.016 |
| ECOG-PS>2, n(%) | 37(24.8) | 27(24.8) | 6(26.1) | 4(23.5) | 1 | 1 | 1 | 1 |
| Ann Arbor Stage III-IV, n(%) | 105(70.5) | 72(66.1) | 20(87.0) | 13(76.5) | 0.115 | 0.25 | 0.566 | 0.566 |
| Extranodal disease, n(%) | 78(52.7) | 50(46.3) | 16(69.6) | 12(70.6) | 0.037 | 0.164 | 0.164 | 1 |
| IPI, n(%) |  |  |  |  | 0.014 | 0.005 | 0.642 | 0.338 |
| Low | 34(22.8) | 31(28.4) | 0(0.00) | 3(17.6) |  |  |  |  |
| Low-intermediate | 31(20.8) | 25(22.9) | 3(13.0) | 3(17.6) |  |  |  |  |
| High-intermediate | 49(32.9) | 32(29.4) | 11(47.8) | 6(35.3) |  |  |  |  |
| High | 35(23.5) | 21(19.3) | 9(39.1) | 5(29.4) |  |  |  |  |
| Double-expressor lymphoma,n(%) | 63(42.3) | 48(44.0) | 10(43.5) | 5(29.4) | 0.521 | 1 | 0.845 | 0.845 |
| Non-GCB subtype, n(%) | 104(69.8) | 72(66.1) | 18(78.3) | 14(82.4) | 0.249 | 0.556 | 0.556 | 1 |
| Treatment response, n(%) | |  |  |  | 0.593 | 0.698 | 0.698 | 1 |
| CR | 108(72.5) | 82(75.2) | 15(65.2) | 11(64.7) |  |  |  |  |
| PD | 30(20.1) | 18(16.5) | 7(30.4) | 5(29.4) |  |  |  |  |
| PR | 10(6.71) | 8(7.34) | 1(4.35) | 1(5.88) |  |  |  |  |
| SD | 1(0.67) | 1(0.92) | 0(0.00) | 0(0.00) |  |  |  |  |

PTA; platelet-to-albumin, IQR; interquartilerange, ECOG-PS; eastern cooperative oncology group performance status, LDH; lactate dehydrogenase, IPI; International Prognostic Index, Extranodal involvement: the bone marrow, CNS, liver/GI tract, spleen, lung and other sites. CR; complete remission, PD; progressive disease, PR; partial response, SD; stable disease.

Table S7. Univariate analysis of the PTA ratio as a categorical variable in the internal validation set

|  | Progression-free survival | | Overall survival | |
| --- | --- | --- | --- | --- |
| Variables | P value | (95% CI) | P value | (95% CI) |
| PTA ratio classifications | |  |  |  |
| **Low vs. Intermediate** | <0.001 | 3.812(2.123,6.846) | <0.001 | 5.403(2.598,11.236) |
| **High vs. Intermediate** | 0.001 | 3.452(1.665,7.157) | <0.001 | 7.342(3.112,17.32) |
| **Male vs. Female** | 0.017 | 1.916(1.122,3.271) | 0.227 | 1.479(0.784,2.789) |
| **IPI** |  |  |  |  |
| **Low-intermediate vs. Low** | 0.015 | 6.593(1.444,30.104) | 0.06 | 7.637(0.918,63.519) |
| **High-intermediate vs. Low** | 0.002 | 9.706(2.281,41.302) | 0.016 | 11.904(1.578,89.802) |
| **High vs. Low** | <0.001 | 21.726(5.129,92.027) | 0.001 | 26.869(3.554,203.113) |
| **GCB vs. Non-GCB** | 0.019 | 2.173(1.139,4.145) | 0.173 | 1.658(0.801,3.429) |
| **DEL(Yes vs.NO)** | 0.344 | 1.284(0.765,2.156) | 0.114 | 1.656(0.886,3.094) |

PTA; platelet-to-albumin, IPI; International Prognostic Index. DEL, double expressor lymphoma, COO; cell-of-origin

Table S8. Multivariate analyses of the PTA as a categorical variable for OS in the internal validation set

|  | Overall survival | |
| --- | --- | --- |
| Variables | P value | (95% CI) |
| PTA ratio |  |  |
| **Low vs. Intermediate** | <0.001 | 4.059(1.891,8.714) |
| **High vs. Intermediate** | <0.001 | 6.987(2.737,17.838) |
| **Male vs. Female** | 0.057 | 2.015(0.98,4.144) |
| **IPI** |  |  |
| **Low-intermediate vs. Low** | 0.079 | 6.746(0.799,56.965) |
| **High-intermediate vs. Low** | 0.064 | 6.942(0.891,54.071) |
| **High vs. Low** | 0.015 | 12.713(1.645,98.241) |
| **GCB vs. Non-GCB** | 0.517 | 1.282(0.605,2.716) |
| **DEL(Yes vs.NO)** | 0.287 | 1.444(0.734,2.842) |

PTA; platelet-to-albumin, IPI; International Prognostic Index, COO; cell-of-origin

Table S9. Univariate analysis of the PTA ratio as a categorical variable in the external validation set

|  | Progression-free survival | | Overall survival | |
| --- | --- | --- | --- | --- |
| Variables | P value | (95% CI) | P value | (95% CI) |
| PTA ratio | |  |  |  |
| **Low vs. Intermediate** | <0.001 | 5.222(2.065,13.21) | 0.025 | 6.382(1.265,32.182) |
| **High vs. Intermediate** | 0.003 | 2.764(1.423,5.37) | 0.024 | 3.635(1.188,11.126) |
| **Male vs. Female** | 0.782 | 1.083(0.614,1.91) | 0.495 | 1.435(0.509,4.044) |
| **IPI** |  |  | 0.001 | 7.66(2.21,26.54)# |
| **Low-intermediate vs. Low** | 0.05 | 7.874(1.004,61.749) |  |  |
| **High-intermediate vs. Low** | 0.019 | 11.719(1.494,91.931) |  |  |
| **High vs. Low** | 0.007 | 15.237(2.075,111.874) |  |  |
| **GCB vs. Non-GCB** | 0.058 | 1.907(0.979,3.715) | 0.103 | 2.814(0.812,9.746) |

PTA; platelet-to-albumin, IPI; International Prognostic Index. COO; cell-of-origin. # indicates the comparison of high vs others of IPI levels

Table S10. Clinical features of DLBCL patients in the external validation set

| Variables | Total patients | Intermediate group(IG) | Low group(LG) | High group(HG) | P values | | | |
| --- | --- | --- | --- | --- | --- | --- | --- | --- |
|  |  |  |  |  | Total | IG vs. LG | IG vs. HG | LG vs HG |
| Number | 110 | 82 | 7 | 21 |  |  |  |  |
| PTA ratio, median[IQR] | 5.39 [4.36;7.08] | 5.16 [4.36;6.06] | 2.15 [1.59;2.23] | 10.5 [9.70;11.4] | <0.001 | <0.001 | <0.001 | <0.001 |
| PLT (10^9/L, median[IQR]) | 215 [161;258] | 204 [161;243] | 80.0 [50.0;87.5] | 368 [322;394] | <0.001 | <0.001 | <0.001 | <0.001 |
| ALB (g/L, median[IQR]) | 37.3 [32.9;40.7] | 38.3 [36.2;41.3] | 36.1 [33.3;40.3] | 32.7 [30.5;37.1] | 0.001 | 0.307 | 0.001 | 0.304 |
| Sex, male, n(%) | 66 (60.0) | 53 (64.6) | 4 (57.1) | 9 (42.9) | 0.176 | 0.699 | 0.35 | 0.699 |
| Age, years | 65.0 [53.0;73.0] | 66.0 [53.0;75.0] | 72.0 [65.5;73.0] | 56.0 [52.0;68.0] | 0.179 | 0.253 | 0.253 | 0.139 |
| LDH (U/L, median[IQR]) | 225 [169;339] | 213 [167;318] | 388 [298;905] | 298 [207;347] | 0.014 | 0.023 | 0.127 | 0.127 |
| ECOG-PS>2, n(%) | 52 (47.3) | 40 (48.8) | 5 (71.4) | 7 (33.3) | 0.199 | 0.434 | 0.434 | 0.309 |
| Ann Arbor Stage III-IV, n(%) | 85 (77.3) | 57 (69.5) | 7 (100) | 21 (100) | 0.001 | 0.184 | 0.017 | 1 |
| Extranodal disease, n(%) | 85 (77.3) | 57 (69.5) | 7 (100) | 21 (100) | 0.001 | 0.184 | 0.017 | 1 |
| IPI, n(%) |  |  |  |  | 0.100 | 0.157 | 0.157 | 0.157 |
| Low | 14 (12.7) | 14 (17.1) | 0 (0.00) | 0 (0.00) | |  |  |  |
| Low-intermediate | 26 (23.6) | 20 (24.4) | 0 (0.00) | 6 (28.6) | |  |  |  |
| High-intermediate | 21 (19.1) | 14 (17.1) | 1 (14.3) | 6 (28.6) | |  |  |  |
| High | 49 (44.5) | 34 (41.5) | 6 (85.7) | 9 (42.9) | |  |  |  |
| Non-GCB subtype, n(%) | 77 (70.0) | 56 (68.3) | 6 (85.7) | 15 (71.4) | 0.812 | 0.990 | 0.990 | 0.990 |
| Treatment response, n(%) |  |  |  |  | 0.001 | 0.005 | 0.039 | 0.255 |
| CR | 76 (69.1) | 64 (78.0) | 1 (14.3) | 11 (52.4) | |  |  |  |
| PD | 11 (10.0) | 6 (7.32) | 2 (28.6) | 3 (14.3) | |  |  |  |
| PR | 9 (8.18) | 3 (3.66) | 2 (28.6) | 4 (19.0) | |  |  |  |
| SD | 14 (12.7) | 9 (11.0) | 2 (28.6) | 3 (14.3) | |  |  |  |

PTA; platelet-to-albumin, IQR; interquartilerange, ECOG-PS; eastern cooperative oncology group performance status, LDH; lactate dehydrogenase, IPI; International Prognostic Index, Extranodal involvement: the bone marrow, CNS, liver/GI tract, spleen, lung and other sites. CR; complete remission, PD; progressive disease, PR; partial response, SD; stable disease.

Table S11. Multivariate analyses of the PTA as a categorical variable in the external validation set

|  | Overall survival | |
| --- | --- | --- |
| Variables | P value | (95% CI) |
| PTA ratio |  |  |
| **Low vs. Intermediate** | 0.296 | 2.43（0.46，12.84） |
| **High vs. Intermediate** | 0.002 | 7.482（2.066，27.097） |
| **Male vs. Female** | 0.129 | 2.374（0.778，7.244） |
| **IPI(High-rsik vs. Others)** | 0.001 | 8.377（2.279，30.789） |
| **GCB vs. Non-GCB** | 0.162 | 2.601（0.682，9.921） |

PTA; platelet-to-albumin, IPI; International Prognostic Index. COO; cell-of-origin


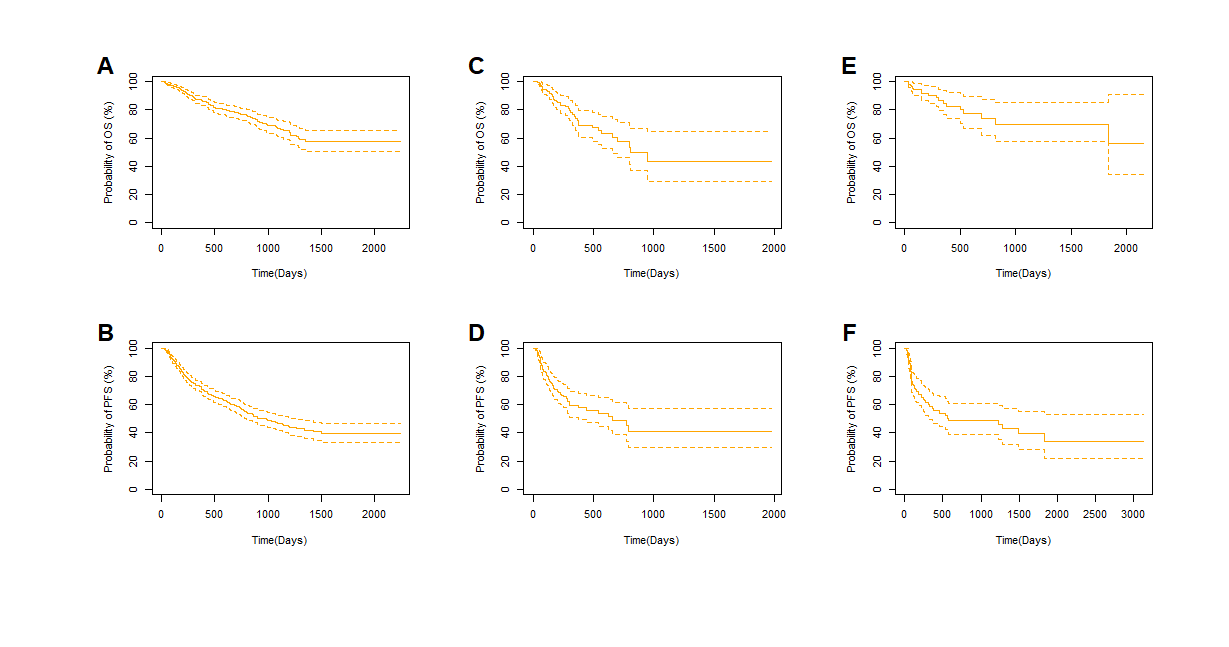


Figure S1. Overall survival (OS) and progression-free survival (PFS) curves were illustrated for patients in the training set(A-B), the internal (C-D) and external validation sets (E-F).


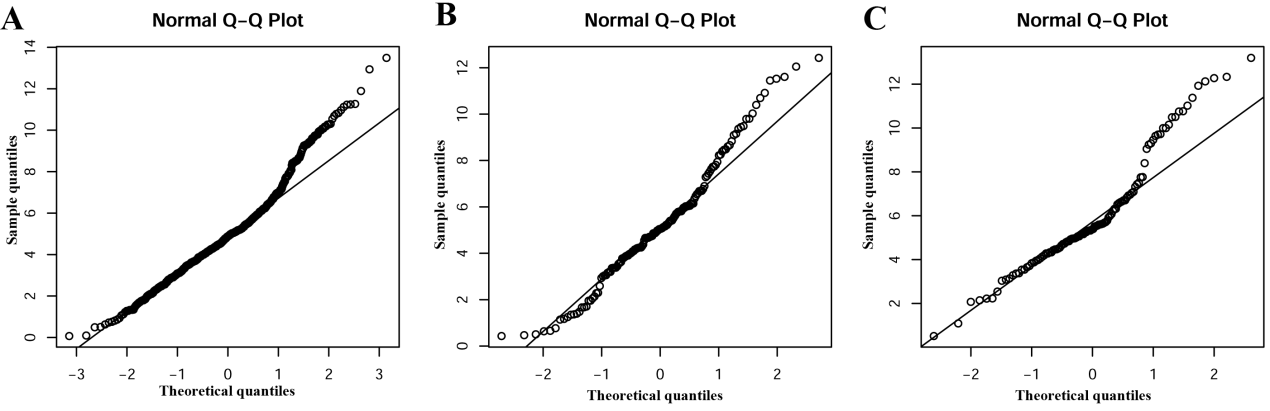


Figure S2. Q-Q plots demonstrated the PTA ratio was non-normal in the training set (A), the internal validation set (B), and the external validation set (C), respectively.


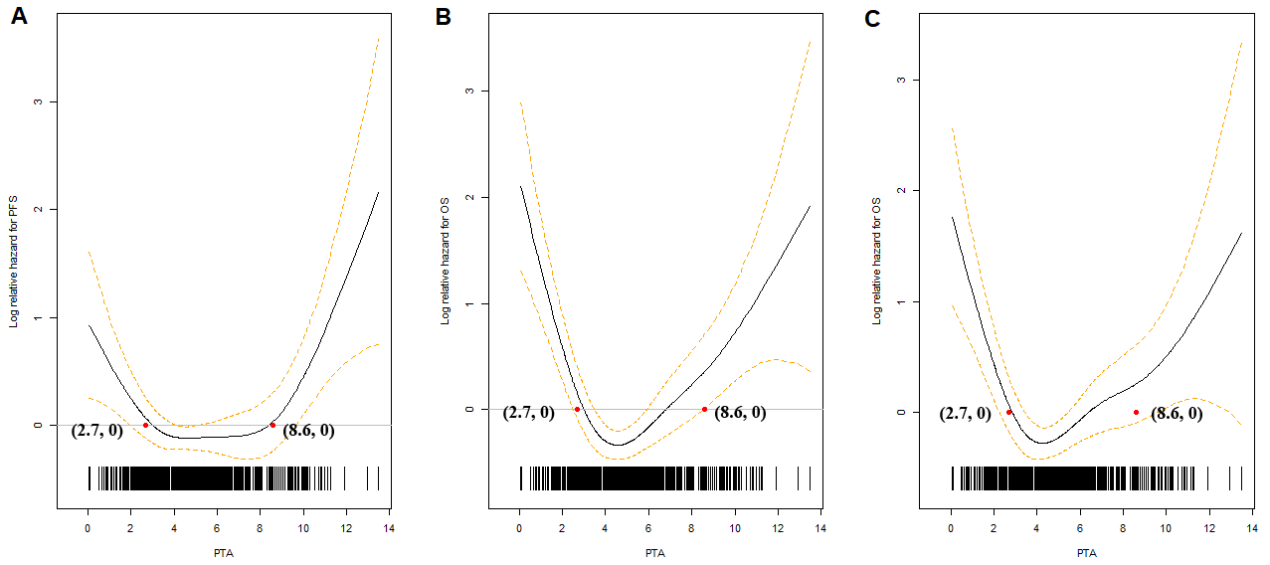


Figure S3. U-shaped relationship between the PTA ratio and PFS(A) after adjusting for IPI, DEL, COO classification, and sex; U-shaped pattern between OS and PTA in the univariate analysis (B), and multivariate analysis (C) after adjusting for IPI, DEL, COO classification, and sex by the penalized smoothing splines models in the training set. Red dots represent the optimal cutoff values of 2.7 and 8.6.


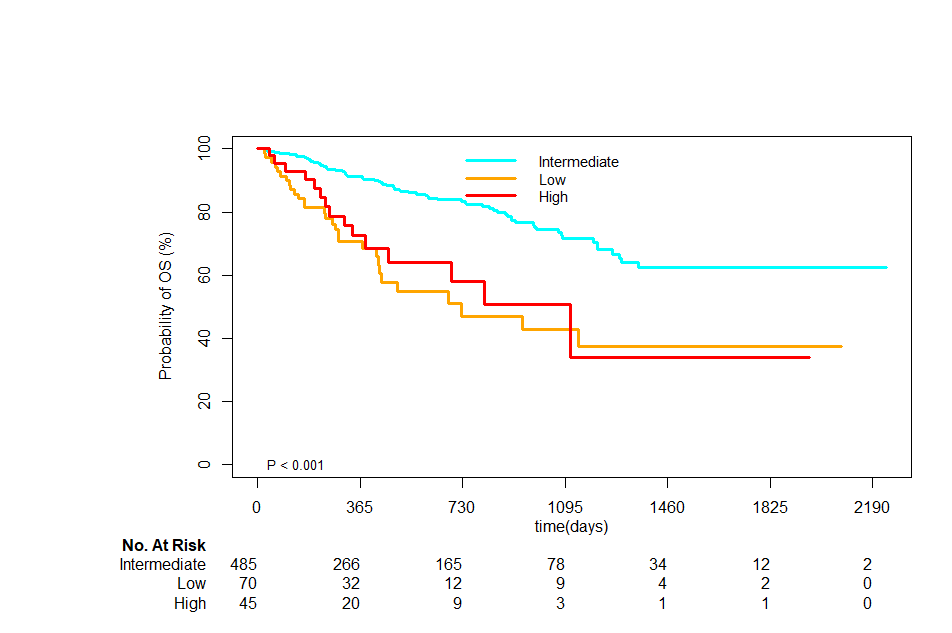


Figure S4. Survival curves for OS by the PTA ratio as a three-categorical variable in the training set


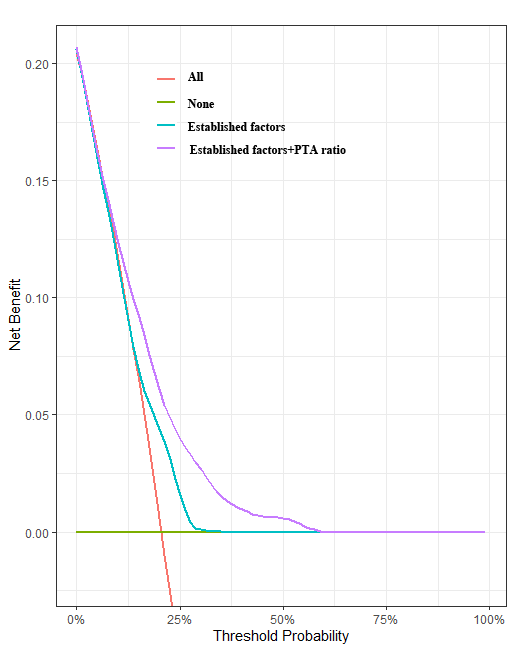


Figure S5. Decision curve analysis showing the clinical utility of the well-established predictors combining sex, COO classification, DEL, and IPI with and without PTA ratio in Cox regression analysis to predict OS in training set. The red straight line represents the net benefit of treating all patients without PTA ratio, assuming that all patients would survive. The green straight line represents the net benefit of treating all patients similarly, assuming that all would die. The cyan line represents the net benefit of treating patients according to the combining the well-established factors including sex, COO classification, DEL, and IPI. The purple line represents the net benefit of treating patients according to the well-established predictors plus PTA ratio.


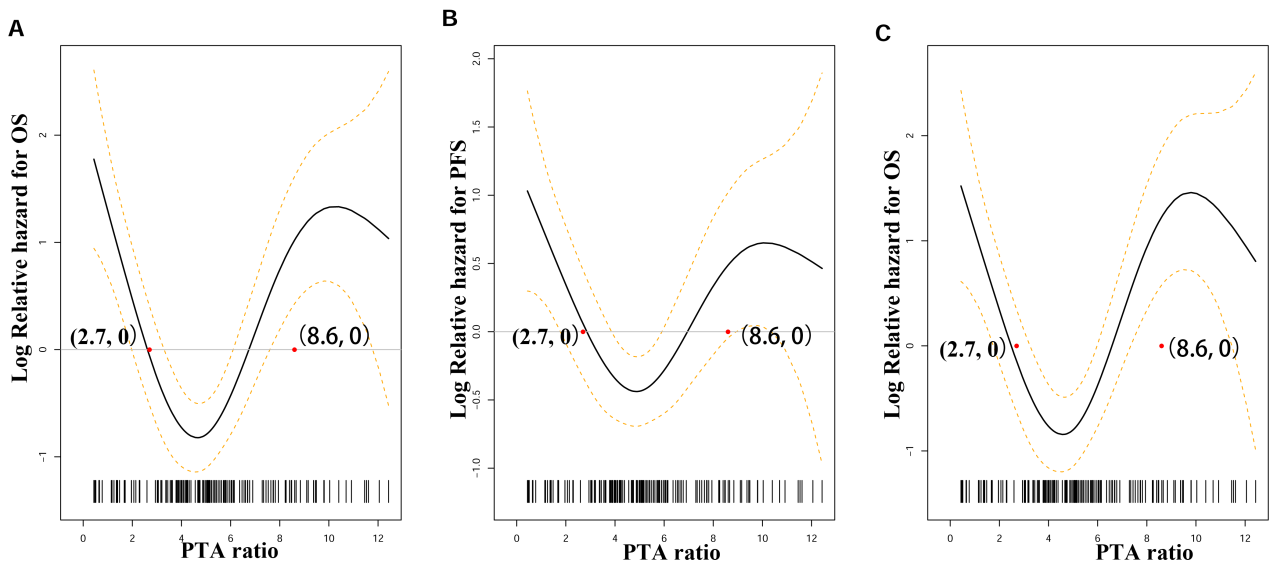


Figure S6. U-shaped relationship between the PTA ratio and OS in the univariate analysis(A), U-shaped relationship between PFS (B) as well as OS(C) and the PTA ratio in multivariate analysis after adjusting for IPI, DEL, COO classification, and sex by the penalized smoothing splines models in the internal validation set. Red dots represent the optimal cutoff values of 2.7 and 8.6.


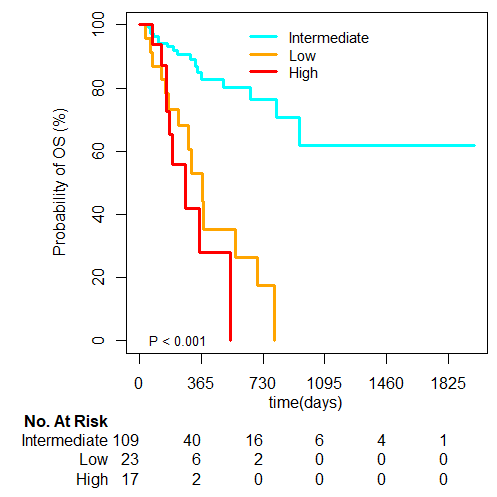


Figure S7. Survival curves of OS by the PTA ratio as a three categorical variable in the internal validation set


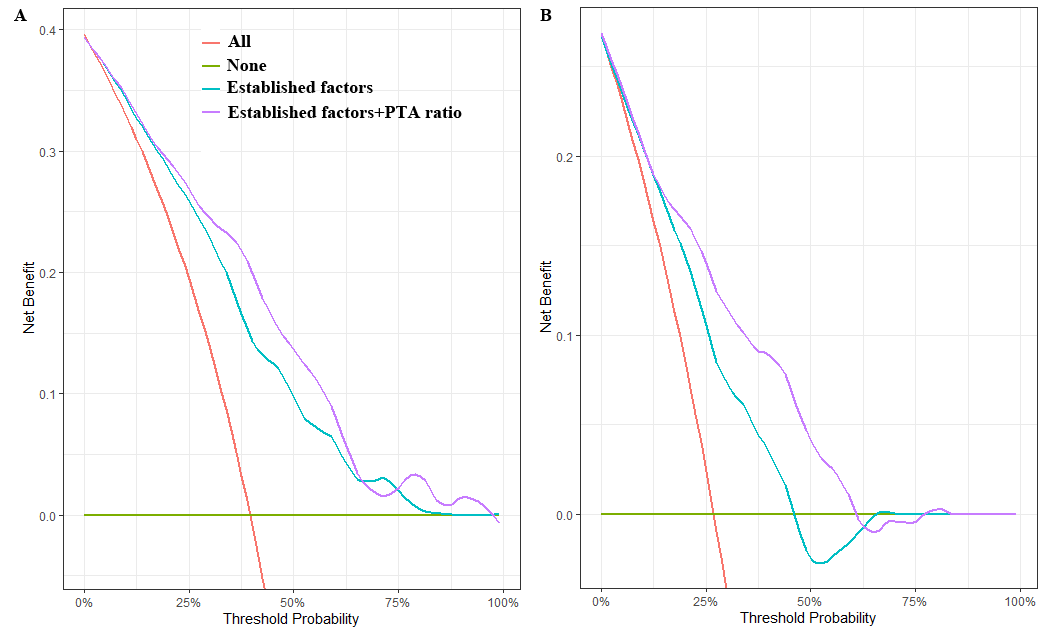


Figure S8. Decision curve analysis showing the clinical utility of the well-established predictors combining sex, COO classification, DEL, and IPI with and without the PTA ratio in Cox regression analyses to predict PFS(A) and OS(B) in 149 patients from the internal validation set. The red straight line represents the net benefit of treating all patients without the PTA ratio, assuming that all patients would survive. The green straight line represents the net benefit of treating all patients similarly, assuming that all would die. The cyan line represents the net benefit of treating patients according to the combining the well-established factors including sex, COO classification, DEL, and IPI. The purple line represents the net benefit of treating patients according to the well-established predictors plus the PTA ratio.


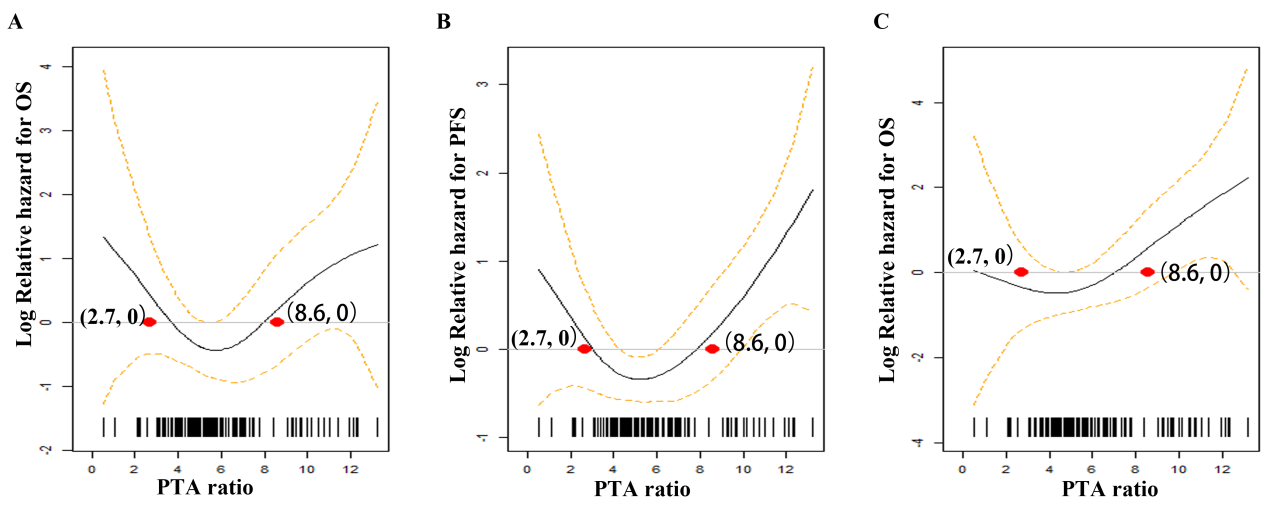


Figure S9. U-shaped relationship between the PTA ratio and OS in the univariate analysis (A); U-shaped pattern between PFS and the PTA ratio in multivariate analysis after adjusting for IPI, COO classification, and sex(B); U-shaped pattern between OS and the PTA ratio disappeared (C) after adjusting for IPI, COO classification, and sex by the penalized smoothing splines models in the external validation set. Red dots represent the optimal cutoff values of 2.7 and 8.6.


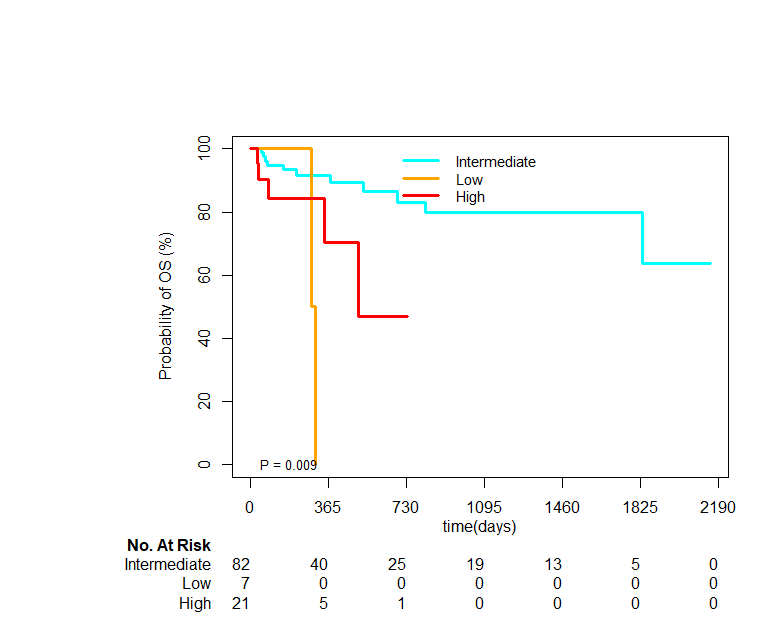


Figure S10. Survival curves of OS by the PTA ratio as a three categorical variable in the external validation set
